# The management of heart failure cardiogenic shock: an international RAND appropriateness panel

**DOI:** 10.1186/s13054-024-04884-5

**Published:** 2024-04-02

**Authors:** Stefan Williams, Antonis Kalakoutas, Segun Olusanya, Benedict Schrage, Guido Tavazzi, Anthony P. Carnicelli, Santiago Montero, Christophe Vandenbriele, Adriana Luk, Hoong Sern Lim, Sai Bhagra, Sascha C. Ott, Marta Farrero, Marc D. Samsky, Jamie L. W. Kennedy, Sounok Sen, Richa Agrawal, Penelope Rampersad, Amanda Coniglio, Federico Pappalardo, Christopher Barnett, Alastair G. Proudfoot

**Affiliations:** 1 Perioperative Medicine Department, Barts Heart Centre, St Bartholomew’s Hospital, West Smithfield, London, EC1A 7BE UK; 2 William Harvey Research Institute, Barts and The London School of Medicine and Dentistry, Queen Mary University of London, London, UK; 3 Department of Cardiology, University Heart and Vascular Center Hamburg, Hamburg, Germany; 4 Department of Clinical-Surgical, Diagnostic and Pediatric Sciences, University of Pavia, Pavia, Italy; 5 Intensive Care, Fondazione Policlinico San Matteo Hospital IRCCS, Pavia, Italy; 6 Division of Cardiology, Medical University of South Carolina, Charleston, SC USA; 7 Acute Cardiovascular Care Unit, Cardiology, Hospital Germans Trias i Pujol, Departament de Medicina, Universitat Autònoma de Barcelona, Barcelona, Spain; 8 Department of Cardiovascular Diseases, University Hospitals Leuven, Leuven, Belgium; 9 Division of Cardiology, Department of Medicine, Peter Munk Cardiac Centre, University Health Network, Toronto, ON Canada; 10 Department of Cardiology, University Hospital Birmingham NHS Foundation Trust, Birmingham, UK; 11 Institute of Cardiovascular Sciences, University of Birmingham, Birmingham, UK; 12 Advanced Heart Failure and Transplantation, Royal Papworth Hospital NHS Foundation Trust, Cambridge, UK; 13 Department of Cardiac Anesthesiology and Intensive Care Medicine, German Heart Center Berlin, Berlin, Germany; 14 Hospital Clinic of Barcelona, Barcelona, Spain; 15 Section of Cardiovascular Medicine, Yale University School of Medicine, New Haven, CT USA; 16 Heart Failure / Transplant Program, Inova Heart and Vascular Institute, Falls Church, VA USA; 17 Division of Cardiology, Duke University Medical Center, Durham, NC USA; 18 Division of Cardiology, Cleveland Clinic Foundation, Cleveland, OH USA; 19 Department of Cardiothoracic and Vascular Anaesthesia and Intensive Care, AO SS Antonio e Biagio e Cesare Arrigo, Alessandria, Italy; 20 Division of Cardiology, Department of Medicine, University of California San Francisco, San Francisco, CA USA

**Keywords:** RAND, Decompensated heart failure, Shock, Cardiogenic, Haemodynamic

## Abstract

**Background:**

Observational data suggest that the subset of patients with heart failure related CS (HF-CS) now predominate critical care admissions for CS. There are no dedicated HF-CS randomised control trials completed to date which reliably inform clinical practice or clinical guidelines. We sought to identify aspects of HF-CS care where both consensus and uncertainty may exist to guide clinical practice and future clinical trial design, with a specific focus on HF-CS due to acute decompensated chronic HF.

**Methods:**

A 16-person multi-disciplinary panel comprising of international experts was assembled. A modified RAND/University of California, Los Angeles, appropriateness methodology was used. A survey comprising of 34 statements was completed. Participants anonymously rated the appropriateness of each statement on a scale of 1 to 9 (1–3 as inappropriate, 4–6 as uncertain and as 7–9 appropriate).

**Results:**

Of the 34 statements, 20 were rated as appropriate and 14 were rated as inappropriate. Uncertainty existed across all three domains: the initial assessment and management of HF-CS; escalation to temporary Mechanical Circulatory Support (tMCS); and weaning from tMCS in HF-CS. Significant disagreement between experts (deemed present when the disagreement index exceeded 1) was only identified when deliberating the utility of thoracic ultrasound in the immediate management of HF-CS.

**Conclusion:**

This study has highlighted several areas of practice where large-scale prospective registries and clinical trials in the HF-CS population are urgently needed to reliably inform clinical practice and the synthesis of future societal HF-CS guidelines.

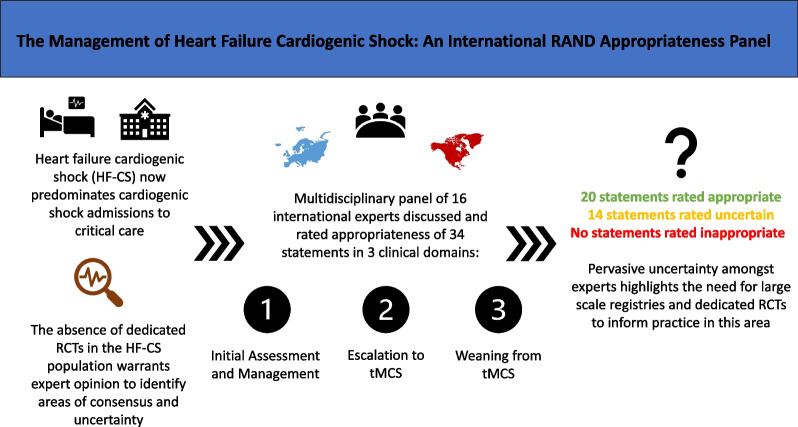

**Supplementary Information:**

The online version contains supplementary material available at 10.1186/s13054-024-04884-5.

## Background

Cardiogenic shock (CS) represents the final common pathway by which cardiovascular disease causes end-organ dysfunction through sustained hypoperfusion and tissue dysoxia [1]. As a clinical syndrome, CS exhibits heterogeneity with respect to its causative aetiologies, presentation, trajectory, and therapeutic responsiveness. As such, CS continues to pose significant diagnostic and therapeutic challenges. This likely contributes to considerable variation of care and the persistently poor clinical outcomes with mortality between 30 and 50% [1–3]. Randomised clinical trial (RCT) enrolment has almost exclusively focussed on the cohort with acute myocardial infarction-related cardiogenic shock (AMI-CS), and the only intervention proven to reduce mortality is coronary revascularisation restricted to the culprit lesion in AMI-CS [1].

Observational data suggest that the subset of patients with heart failure related CS (HF-CS) now predominate CS admissions to critical care [2–4]. HF-CS is broadly subcategorized into *de novo* HF-CS wherein acute decompensated HF (ADHF) causing CS is identified in the absence of a prior HF diagnosis, and *acute-on-chronic* HF-CS where ADHF leads to CS development in context of a pre-existing HF diagnosis [5, 6]. This shift in epidemiology of CS towards a preponderance of critical care admissions with HF-CS appears to be driven by a greater relative increase in cases of new-onset or chronic cardiomyopathy with decompensated heart failure as compared with acute myocardial infarction [1, 4, 11]. This has implications for clinical management and service design; recent single centre data have demonstrated that compared with AMI-CS patients HF-CS are younger, are less likely to have cardiac arrest, have divergent haemodynamic profiles, have different requirements for temporary mechanical circulatory support (tMCS) and a different clinical course with lower in-hospital mortality [13]. Current clinical guidelines do not reflect these emerging differences and there are no dedicated HF-CS RCTs completed to date to reliably inform clinical practice.

In an effort to consolidate opinion around the management of HF-CS, we conducted a modified Delphi consensus process using modified RAND/University of California, Los Angeles, appropriateness methodology. We specifically focused on the subset of patients with an acute decompensation of chronic HF given the heterogeneity of aetiologies and potential for disease specific management in the *de novo* cohort [7]. Our intent was to identify aspects of care where both consensus and uncertainty may exist to inform clinical practice and to focus efforts for future clinical trial design.

## Methods

The RAND/UCLA appropriateness method (University of California, Los Angeles) utilises a modified Delphi panel approach to collate expert opinion, based on clinical expertise and available evidence, to determine the appropriateness of clinical decisions in clearly defined clinical scenarios (https://www.rand.org/topics/methodology.html) [5]. This methodology is particularly useful when examining areas in which practice is uncertain, or evidence is lacking, insufficient or in disagreement. It is validated to determine the benefit or harm of an intervention irrespective of cost, resources and timing; to identify best possible practice.

A literature search was conducted, to identify all prior publications relating to HF-CS since January 2017 (Additional file 1: Figure 1). This was forwarded as a bibliography, along with a web-based questionnaire designed and iterated by a core group (Proudfoot, Tavazzi, Pappalardo, Samsky) to a panel of 16 experts across in advanced heart failure cardiology, cardiac intensive care and interventional cardiology. Experts were identified through international meetings, publications in the field and selected from a range of countries to encapsulate potential variable practice in HF-CS across healthcare systems; as such, they were representing themselves with no societal or commercial affiliation. Institutional review board approval was waived given the nature of the study. The panellists were asked to utilise their clinical expertise, with the support of the supplementary bibliography, to rate the appropriateness of specific management options via the online questionnaire. They were asked to rate the interventions on a scale of 1 to 9, in ascending order of appropriateness, whereby 1 to 3 is inappropriate, 4 to 6 is uncertain and 7 to 9 being appropriate.

The questionnaire comprised questions, subdivided into the following 3 sections; the initial assessment and management of HF-CS (10 questions); escalation to tMCS (17 questions); weaning from tMCS in HF-CS (7 questions).

The clinical scenarios were based on several assumptions. All patients were assumed to have chronic heart failure with decompensation as defined by recent consensus definition [8], and not *de novo*, acute myocardial infarction, post-cardiotomy or cardiac arrest related cardiogenic shock. Decisions on appropriateness were based on the clinical scenario presented alone. We chose to focus on Society for Cardiovascular Angiography and Interventions (SCAI) stage C patients (patients with clinical evidence of hypoperfusion initially requiring pharmacological or mechanical support in the escalation component of the survey as this is the most prevalent cohort in contemporary registries [9–12].

The responses of the questionnaire were summarised and anonymised with aliases, and presented in a virtual meeting, moderated by an expert in the methodology but non-expert in HF-CS. The moderator remained neutral throughout. During the meeting, the questions and clinical scenarios were reviewed to ensure clarity. The results instigated discussion, with a focus placed on areas of disagreement. Consensus was not sought, as uncertainty is a valid outcome. The discussion was scribed to support write-up. Following the meeting, the online questionnaire was re-sent to all participants. The questionnaire was then re-completed by each participant with modification of their original, pre-panel response, based on the discussions. This score was the final score used for analysis.

For each scenario, median scores were calculated with a score of <3.5 being considered inappropriate, ≥3.5 and <6.5 uncertain, and ≥6.5 appropriate. The validated RAND disagreement index (DI) was calculated to define disagreement (DI ≥ 1) amongst panellists using the equation below [13]. Any scenario in which disagreement was found was scored as uncertain, regardless of the median score.

$$DI = \frac{70\% ile - 30\% ile}{{\begin{array}{*{20}c} {2.35 + \left( {1.35 \times abs\left( {5 - \frac{70\% ile + 30\% ile}{2}} \right)} \right)} \\ \end{array} }}$$

## Results

**Fig. 1:**
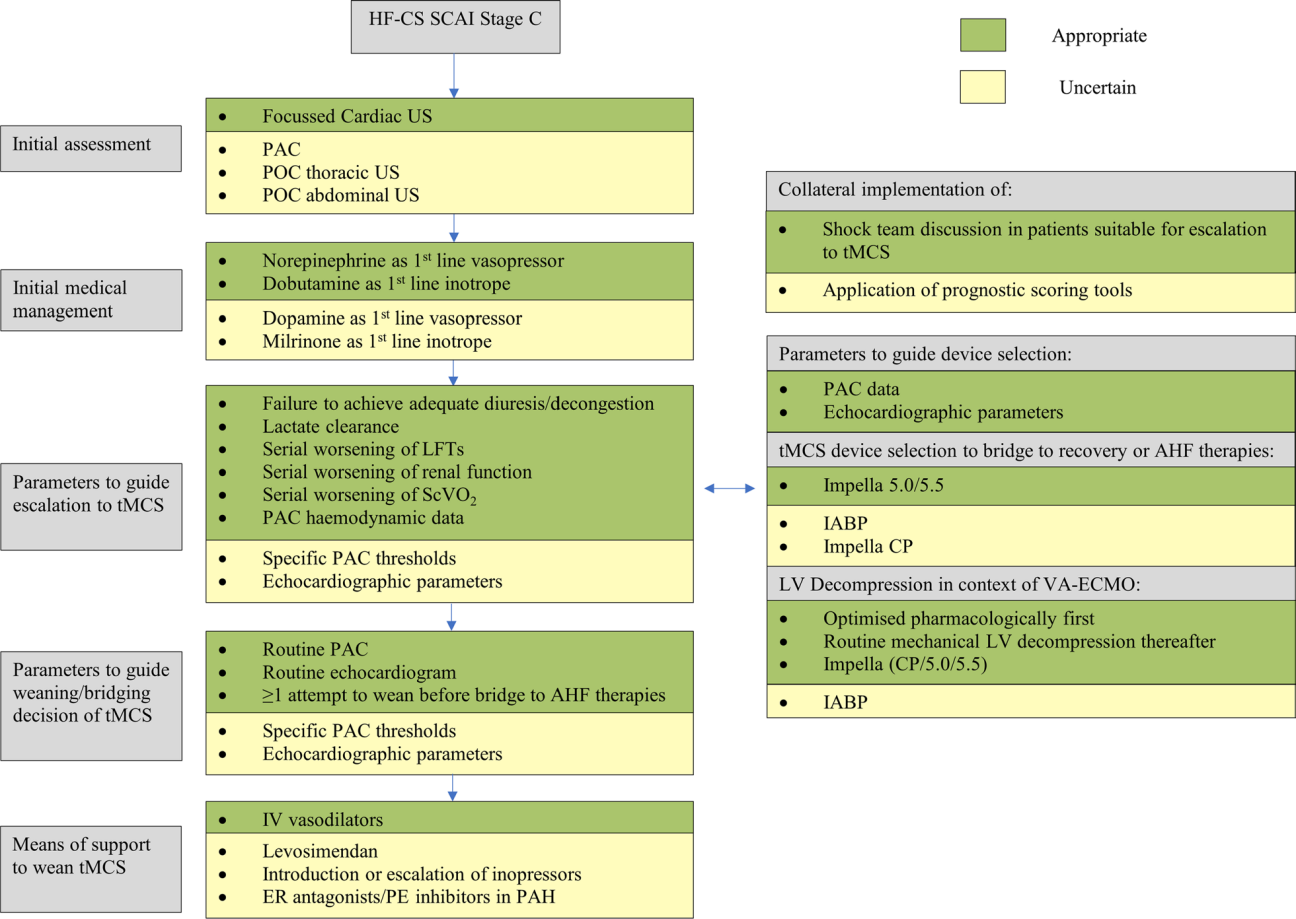
Management algorithm summarizing RAND panel recommendations in heart failure cardiogenic shock (HF-CS). AHF, Advanced Heart Failure; CP, Central Pump; ER, Endothelin Receptor; HF-CS, Heart Failure related Cardiogenic Shock; IABP, Intraaortic Balloon Pump; IV, Intravenous; LFT, Liver Function Tests; LV, Left Ventricle; PAC, Pulmonary Artery Catheterisation; PAH, Pulmonary Arterial Hypertension; PE, Phosphodiesterase Inhibitor; POC, Point-of-Care; SCAI, Society of Cardiovascular Angiography and Interventions; ScVO2, Systemic central Venous Oxygen Levels; tMCS, temporary Mechanical Circulatory Support; US, Ultrasound.

20 of the statements were rated as appropriate and 14 as uncertain. None of the statements was rated as inappropriate (Tables 1, 2, 3). Figure 1 categorises statements based on their respective clinical domain, as well as illustrating panellist determined appropriateness. Anonymised individual panellist scoring is outlined in Additional file 1: Tables S1 through S3. One of the statements had a disagreement index of >1 (Table 1), representing statistically significant disagreement and no clinical equipoise reached based on the study methodology.

**Table 1:** The initial assessment and management of HF-CS

Statements	Median	Disagreement index (DI)	Inter-percentile range (IPR)	RAND panel outcome
*Please rate the appropriateness of the following in the initial assessment and management of SCAI Stage C HF-CS:*				
Focussed Cardiac Ultrasound	9	0.13	1.00	Appropriate
Pulmonary artery catheter	6	0.51	2.00	Uncertain
Point of care thoracic ultrasound	5	1.27	4.00	Uncertain
Point of care Abdominal Ultrasound	3.5	0.35	2.25	Uncertain
Norepinephrine as 1^st^ line vasopressor	7	0.16	1.00	Appropriate
Dopamine as 1^st^ line vasopressor	3.5	0.51	2.25	Uncertain
Dobutamine as 1^st^ line inotrope	6.5	0.35	2.25	Appropriate
Milrinone as 1^st^ line inotrope	6	0.51	2.00	Uncertain
Shock team discussion in patients suitable for escalation to tMCS	8	0.29	2.00	Appropriate
Application of prognostic scoring tools e.g. IHVI and CardShock to inform management and escalation	4	0.51	2.00	Uncertain

For each question, median scores were allocated as inappropriate if scoring <3.5, uncertain if ≥3.5 and <6.5 uncertain and appropriate if ≥6.5. DI was calculated using the RAND DI and disagreement deemed if DI ≥1 amongst the panellists.

HF-CS, Heart Failure related Cardiogenic Shock; IHVI, Inova Heart and Vascular Institute; SCAI, The Society of Cardiovascular Angiography and Interventions; tMCS, temporary Mechanical Circulatory Support

**Table 2:** Escalation to tMCS in HF-CS

Statements	Median	Disagreement index (DI)	Inter-percentile range (IPR)	RAND panel outcome
*Regarding the use of clinical, biochemical and haemodynamic parameters to guide escalation to tMCS in the context of maximal or optimal pharmacotherapy, please rate the appropriateness of the following:*				
Failure to achieve adequate diuresis/clinical decongestion	7.5	0.16	1.25	Appropriate
Lactate clearance	8.0	0.07	1.25	Appropriate
Serial worsening of liver function tests (bilirubin, transaminases & INR)	7.5	0.16	1.25	Appropriate
Serial worsening of renal function (urine output, creatinine, eGFR)	7.0	0.26	2.00	Appropriate
Serial worsening of central venous oxygen saturations (ScVO_2_)	7.0	0.26	2.00	Appropriate
PAC haemodynamic data to inform escalation decisions	7.5	0.43	3.00	Appropriate
PAC haemodynamic data to inform device selection	8.0	0.23	2.25	Appropriate
Specific PAC thresholds (informed by AHA guidance [41], Geller et al. [42]) to inform escalation decisions	4.5	0.55	2.25	Uncertain
Echocardiographic parameters to guide escalation decisions	6	0.43	2.25	Uncertain
Echocardiographic parameters to guide device selection	7	0.37	2.25	Appropriate
*Regarding the selection of tMCS in the management of SCAI Stage C HF-CS, please rate the appropriateness of the following:*				
IABP as a tMCS option for bridge to recovery or durable therapies	5.5	0.71	3.00	Uncertain
Impella™ CP as a tMCS option for bridge to recovery or candidacy for durable HF therapies	5.0	0.55	2.25	Uncertain
Impella™ 5.0/5.5 as a tMCS option for bridge to recovery or candidacy for AHF therapies	7.0	0.21	1.25	Appropriate
Routine mechanical LV decompression in the context of peripheral VA ECMO	6.5	0.59	3.25	Appropriate
Optimised pharmacological LV decompression prior to mechanical LV decompression	7.0	0.37	2.00	Appropriate
IABP as a mechanical LV decompression strategy in peripheral V-A ECMO	5.5	0.32	1.25	Uncertain
Impella™ (CP/5.0/5.5) as a mechanical LV decompression strategy in peripheral V-A ECMO	6.5	0.30	2.00	Appropriate

For each question, median scores were allocated as inappropriate if scoring <3.5, uncertain if ≥3.5 and <6.5 uncertain and appropriate if ≥6.5. DI was calculated using the RAND DI and disagreement deemed if DI ≥1 amongst the panellists.

AHA, American Heart Association; AHF, Advanced Heart Failure; eGFR, estimated Glomerular Filtration Rate; HF, Heart Failure; HF-CS, Heart Failure related Cardiogenic Shock; IABP, Intra-aortic Balloon Pump; Impella™ CP, Impella™ Central Pump; INR, International Normalised Ratio; LV, Left Ventricle; PAC, Pulmonary Artery Catheterisation; SCAI, Society for Cardiovascular Angiography and Interventions; ScVO_2_, Systemic Central Venous Oxygen Levels; tMCS, temporary Mechanical Circulatory Support; V-A ECMO, Venoarterial Extracorporeal Membrane Oxygenation

**Table 3:** Weaning from tMCS in HF-CS

Statements	Median	Disagreement index (DI)	Inter-percentile range (IPR)	RAND panel outcome
*Regarding the weaning of tMCS in HF-CS, please rate the appropriateness of the following:*				
Routine PAC to assess / support weaning of tMCS	7.0	0.08	1.00	Appropriate
At least one attempt to wean tMCS before decision to transition to AHF therapies	7.5	0.23	2.00	Appropriate
Routine echocardiogram to assess / support weaning of tMCS	7.0	0.16	1.25	Appropriate
Use of Levosimendan to support weaning of tMCS	4.5	0.59	3.25	Uncertain
Use of escalating inotropes to wean from tMCS	6.0	0.35	2.00	Uncertain
Use of intravenous vasodilators to support weaning from tMCS	6.5	0.35	2.00	Appropriate
Trial of endothelin receptor antagonists or phosphodiesterase inhibitors in patients with evidence of pulmonary hypertension to support weaning from tMCS	5.0	0.43	2.25	Uncertain

For each question, median scores were allocated as inappropriate if scoring <3.5, uncertain if ≥3.5 and <6.5 uncertain and appropriate if ≥6.5. DI was calculated using the RAND DI and disagreement deemed if DI ≥1 amongst the panellists.

HF-CS, Heart Failure related Cardiogenic Shock; PAC, Pulmonary Artery Catheterisation; tMCS, temporary Mechanical Circulatory Support; VAD, Ventricular Assist Device

### The initial assessment and management of SCAI stage C HF-CS

During the initial assessment of SCAI Stage C HF-CS, it was deemed appropriate to perform a focussed cardiac ultrasound and to initiate a shock team discussion in patients that were deemed eligible for escalation to tMCS. Pulmonary Artery Catheterisation (PAC) was considered an uncertain modality for advanced haemodynamic assessment. Point of care Abdominal and Thoracic Ultrasound were also rated as uncertain, with the latter being the only statement whereby there was statistically significant disagreement (DI >1); the median score from European panellists was 6 whereas it was 3 from North American clinicians (Additional file 1: Table S4).

The use of norepinephrine as a 1^st^ line vasopressor and dobutamine as a 1^st^ line inotrope were deemed appropriate. Dopamine and milrinone were deemed uncertain as 1^st^ line agents. The utility of established prognostic scoring systems such as the IHVI [14] and CardShock [15] scores to inform management and escalation was deemed uncertain due to, at best, modest discrimination.

### Escalation to tMCS

Failure to achieve adequate diuresis/decongestion, lactate clearance, serial worsening of liver and renal function tests as well as serial worsening of central venous oxygen saturations (ScVO_2_) were all deemed important parameters to guide escalation to tMCS when HF-CS patients were otherwise optimally medically managed. Following immediate stabilisation, PAC haemodynamic data was deemed to be appropriate both to inform escalation decisions and device selection, whereas echocardiographic parameters were only considered appropriate to guide device selection. However, the value of published measured and derived haemodynamic thresholds to inform escalation decisions [16] were deemed uncertain.

Regarding tMCS modalities as options for bridge to native heart survival or candidacy for heart replacement therapies (HRT), Impella™ 5.0/5.5 (Abiomed, Danvers, Massachusetts) was the only tMCS deemed appropriate. Overall, implantation of the intra-aortic balloon pump (IABP) and Impella™ CP were both considered uncertain. There was a greater than 2-point difference between median responses between experts from Europe compared to North America regarding the use of IABP as a bridge to HRT with clinicians from North America demonstrating more certainty around its utility (Additional file 1: Table S4). Indeed, IABP was the most common MCS device in HF-CS patients in a contemporary North American registry, particularly amongst patients receiving HRT [17]. This practice may reflect alterations to the heart transplant allocation system in the US as well as observational data suggesting IABP may support either bridge to recovery or to HRT in select HF-CS patients [18].

It was deemed appropriate to initially adopt a pharmacological approach with optimal inotropy to facilitate left ventricular (LV) unloading in the context of peripheral venoarterial extracorporeal membrane oxygenation (V-A ECMO) in HF-CS. If tMCS was deployed for LV unloading, there was consensus that the Impella™ family of devices (CP/5.0/5.5) was the most appropriate strategy whilst there was uncertainty regarding the role of IABP in this context.

### Weaning from tMCS in heart failure related cardiogenic shock

Both PAC and echocardiography were deemed appropriate methods of assessing readiness for and guiding weaning of tMCS. Regarding transition to HRT from tMCS, there was broad agreement that there should be at least one attempt to wean tMCS prior to making a formative decision.

Use of intravenous vasodilators was deemed appropriate in supporting weaning from tMCS, especially in the context of elevated right ventricular pressures. Statements on the use of levosimendan and on escalation of inotropes to wean from tMCS were considered uncertain. The use of endothelin receptor antagonists or phosphodiesterase inhibitors in patients with evidence of pulmonary arterial hypertension to support weaning from tMCS was deemed uncertain.

## Discussion

Responses of the panel to the final survey suggested that despite an absence of societal guideline or randomised trial data to inform practice there were many aspects of care where there was alignment of approach. There was very little disagreement between experts; the DI was significant (DI >1) only when addressing the role of thoracic ultrasound in the immediate management of HF-CS. Nonetheless, there remains considerable equipoise as evidenced by just under half (16/34) of statements being rated as uncertain (Fig. 1).

A priori, the expert panel agreed that the management of HF-CS required different approaches and considerations to that of AMI-CS. This reflected clinical observations: 1) pulmonary, venous and visceral congestion were more prevalent at baseline in HF-CS; 2) patients are often ambulatory despite profound derangements in measured cardiac output, cardiac filling pressures and transpulmonary pressure gradients; 3) markers of hypoperfusion, such as capillary refill time, elevation of lactate or derangements of organ function are often comparatively preserved in patients with HF-CS [11]. The initial supportive management of HF-CS is to restore organ perfusion to mitigate progression towards multi-organ failure. This may or may not require normalization of haemodynamics. Concordant with societal guidance [19, 20], panellists agreed that norepinephrine is an appropriate first line vasopressor with uncertainty surrounding the use of dopamine. Dobutamine as the first line inotrope was deemed appropriate whilst milrinone as first line was uncertain. These data align with a recent international survey of CS treatment strategies across 60 countries [21]. RCT data demonstrated no significant difference in a composite outcome including death, cardiac arrest and receipt of tMCS between milrinone and dobutamine in a cohort with mixed aetiology CS [22]. Nonetheless, experts noted that the divergent half-lives of dobutamine and milrinone impacted inotrope selection, particularly in the cohort with more profound hypotension. Of note, recent RCT data suggested no increased dysrhythmia with dobutamine therapy nor greater hypotension with milrinone [22]. There remains low certainty that inotropes *per se* offer benefit and may cause harm. The CAPITAL DOREMI 2 RCT (ClinicalTrials.gov Identifier: NCT05267886) will assess the efficacy and safety of inotropes (dobutamine and milrinone) compared to placebo in all-cause SCAI Stage C/D CS. Imaging with point of care echocardiography in the initial assessment of patients with SCAI stage C HF-CS was deemed appropriate, in alignment with consensus guidance [6, 7]. Routine use of thoracic point of care ultrasound was the only area of statistical disagreement (DI>1). Thoracic ultrasound is a rapid, bedside tool that may have high sensitivity and specificity for identifying the severity of pulmonary congestion which may add diagnostic and prognostic value [23] but panellists deemed that any uplift in diagnostic or therapeutic certainty beyond thorough clinical examination was limited and that widespread use was limited by a lack of training, particularly in North America. The role of PAC and haemodynamic profiling in the initial assessment of CS was similarly deemed uncertain. Immediate classification of HF-CS into biventricular, LV, or right ventricular dominant haemodynamic phenotypes may have therapeutic implications including choice of inopressor or modality of tMCS and confer prognostic value [24] but experts opined that such classification could be derived from routine clinical and biochemical parameters without the potential management delays and complications associated with immediate PAC placement. Recent data in a HF-CS population demonstrated that PAC use was associated with lower in-hospital mortality and early (<6 hours) placement was associated with a more pronounced reduction in mortality compared to either delayed (>48 hours) or no PAC [25]. Of note, and consistent with the panel recommendations below, there was no clear signal of mortality benefit with PAC placement between 6 and 48 hours. This uncertainty will be formally tested by the Pulmonary Artery Catheter in Cardiogenic Shock Trial (PACCS ClinicalTrials.gov Identifier: NCT05485376).

The expert panel were unanimous in recommending that therapeutic response should be guided by serial as opposed to single measures across a broad range of clinical, biochemical and haemodynamic indices to guide therapeutic response and inform trajectory. Appropriate indices included: failure to achieve a target diuresis or decongestion despite maximal diuretics; delayed or impaired lactate clearance; worsening of liver and renal function tests; and a decline in central (or mixed) venous oxygen saturations. This approach is supported by the recent update to the SCAI CS classification which acknowledged that CS is a dynamic condition and advocated repeated reclassification of patients to identify both recovery and deterioration [26]. Aggregate assessments via serial SCAI classification is an independent predictor of mortality [10] and patients who reach SCAI stage E at any stage during their hospital stay have at least a 2-fold increase in mortality compared to those who reached a maximum SCAI Stage of C or D [27]. Extending this further, phenotyping patients based on clinical variables on admission classified by a machine learning approach, enriched the prognostic accuracy of SCAI classification, identified a cohort likely to progress towards SCAI stage E and highlighted an association between phenotype and tMCS device [28]. The use of alternative composite prognostic risk scores, such as the IHVI shock [14] and CardShock [15] scores, was deemed uncertain. Panellists felt that such scores may objectify severity, specifically potential futility, but lacked specificity and validation in the HF-CS cohort. Such scoring systems were, however, advocated to support stratification of patients for enrolment in clinical trials and to facilitate national benchmarking of care.

Consistent with recent observational data, there was consensus that continuous haemodynamic data with PAC should, in combination with the aforementioned clinical and biochemical parameters, inform ongoing management and specifically escalation decisions to tMCS as early as was clinically indicated and feasible. Nonetheless, the utility of specific measured and derived haemodynamic thresholds including cardiac power output and pulmonary artery pulsatility index as indices to guide escalation to tMCS [24] was deemed uncertain. As with all CS aetiologies, HF-CS has a heterogenous clinical presentation and trajectory [29], with sub-phenotypes which may reflect the host response [30, 31], hepatorenal congestion [32] or secondary sepsis. As such, the notion of standardised management guided by haemodynamic cut-off values in such a heterogenous syndrome was deemed to be challenging. The use of serial echocardiographic data to guide escalation was deemed uncertain predominantly due resource and time constraints required but was deemed appropriate to guide tMCS device selection.

Using propensity matching, tMCS use in HF-CS has been associated with a 24% relative risk reduction in 30-day mortality [33] suggesting that there may be differential responses to tMCS in different CS cohorts. The role of IABP as the initial tMCS modality in SCAI C HF-CS as a bridge to recovery or HRT was uncertain. Registry data demonstrate continued use of the IABP across all SCAI stages of HF-CS [10, 11, 28]. Conceptually, the differing physiology of HF-CS with volume and pressure overload as opposed to acute contractile dysfunction may result in comparatively greater afterload reduction and improved organ perfusion [34], a hypothesis supported by clinical data [27, 28]. The role of IABP in the early management of HF-CS compared to vasoactive therapies is currently being assessed in a prospective RCT with a primary end-point of 60-day survival or successful bridge to HRT [35] (ClinicalTrials.gov Identifier: NCT04369573). The Impella™ CP and 5.0/5.5 (Abiomed, Danvers, MA) devices are appealing because they offer direct and more efficient LV unloading compared to IABP. The surgically placed transvalvular Impella™ 5.0/5.5 (Abiomed) was the only tMCS deemed appropriate as a bridge to recovery or HRT. Panellists regarded the greater flow rates of up to 6L/min as being conducive to support of both LV predominant and bi-ventricular failure particularly in those patients with HF-CS supported as a bridge to HRT. Large-scale registry data describing the use and outcome of all tMCS modalities are needed to inform the design of future RCTs in this cohort.

V-A ECMO as a primary support modality in the context of SCAI C HF-CS was not discussed as both European and North American guidance propose that it should be broadly reserved for severe shock states i.e. SCAI D and E [36, 37]. Nonetheless, observational data suggest V-A ECMO continues to be deployed across all severities of CS and patients with HF-CS may be more vulnerable to the associated complications of increased afterload with V-A ECMO due to higher filling pressures at baseline and potential preload reserve exhaustion [38]. Accordingly, the panel deemed that both pharmacological augmentation of native contractility (first line) and then mechanical LV decompression to be appropriate interventions. LV decompression with IABP was deemed inappropriate whilst use of the Impella™ CP and 5.0/5.5 were deemed appropriate. Patient selection and timing for LV decompression remains challenging and has recently been comprehensively reviewed [38]. The UNLOAD-ECMO trial (ClinicalTrials.gov Identifier: NCT05577195) will assess the benefit of LV unloading with Impella™ compared to no unloading across all aetiologies of CS.

Perhaps the most under-studied area of HF-CS management is weaning from tMCS. Responses of the expert panel have reinforced the need for clinical trials in this arena. Beyond combined use of continuous haemodynamic data via PAC and echocardiography to inform and guide suitability for and timing of de-escalation from tMCS there was limited certainty in the optimal approaches, specifically around the use of pharmacotherapy. Despite a recent consensus statement supporting the use of low-dose inopressors to facilitate tMCS weaning [16] the expert group were uncertain regarding this approach; whilst liberation from tMCS mitigates exposure to associated complications, the need for inopressors to support weaning may indicate incomplete or inadequate cardiac recovery and the need for alternative bridging strategies. The use of intravenous vasodilators such as sodium nitroprusside to reduce LV afterload was felt to be an appropriate intervention. Levosimendan, a calcium channel sensitizer, has shown promise with meta-analyses of all-cause CS suggesting improved rates of liberation from V-A ECMO [39, 40]. Panellists were however uncertain regarding its use due to its lack of approval in certain jurisdictions and prolonged half-life. Similarly, there was uncertainty regarding the role of pulmonary vasodilators to facilitate tMCS weaning, including patients with evidence of pulmonary hypertension. These uncertainties highlight the unmet need for both registry and ideally trial data around tMCS weaning strategies.

One of the strengths of our study was the diversity of experts drawn from a range of countries and backgrounds with experience in managing patients with HF-CS. Conversely, the experts were exclusively from Europe and North America; the lack of a truly global perspective may limit the generalizability of our findings albeit that responses are independent of resource. RAND methodology is validated as a guide to decision-making in the absence of a robust evidence base. Whilst the expert panel was provided with a contemporary review of the literature, it was not possible to determine the extent to which knowledge of this influenced responses. Similarly, it was impossible for our scenarios to encompass all cases encountered in clinical practice, and some detailed interventions (e.g. echocardiography) were poorly defined. Discussing the impact of investigations and interventions in isolation is not consistent with clinical practice where multimodal testing is applied. These outcomes should complement existing guidance and inform areas of practice ripe for future clinical trials. Three of the expert panel helped design the original survey; hence, this may have introduced an element of bias; conversely, in accordance with RAND methodology, the nature of the final questionnaire was changed from the original by the entire panel and moderator after the online meeting.

## Conclusions

In conclusion, we have used a multi-professional consensus process that systematically and quantitatively combined expert opinion with the limited available evidence to further inform contemporary management of HF-CS. The preponderance of HF-CS cases in cardiac critical care units coupled with the persisting ambiguity around optimal monitoring, management and escalation of these cases highlighted within, mandates a requirement for both prospective large-scale registries and clinical trials in patients with HF-CS to inform future clinical guidelines. Without this, there is risk that clinicians conflate observational data and guidance generated in the AMI-CS cohort, and as a result, clinical outcomes will remain static. Collaboration between heart failure cardiology, critical care cardiology and trial methodologists is crucial to design trials specific to the HF-CS cohort.

### Supplementary Information

**Additional file 1.** **Figure S1.** PRISMA Flow Diagram of Literature Search. **Table S1.** Anonymised Individual Panellist Scoring: Initial Assessment and Management of HF-CS. **Table S2.** Anonymised Individual Panellist Scoring: Escalation to tMCS in HF-CS. **Table S3.** Anonymised Individual Panellist Scoring: Weaning of tMCS in HF-CS. **Table S4.** Median Scores by Geographical Location (Europe vs North America).

## Data Availability

Not applicable.

## Abbreviations

CS: Cardiogenic shock
HF-CS: Heart failure cardiogenic shock
HRT: Heart replacement therapies
PAC: Pulmonary artery catheter
SCAI: Society of cardiovascular angiography and interventions
tMCS: Temporary mechanical circulatory support
V-A ECMO: Veno-arterial extracorporeal membrane oxygenation
