# Supplementary material for: The management of heart failure cardiogenic shock: an international RAND appropriateness panel
Source: Crit Care. 2024 Apr 2;28:105. doi: 10.1186/s13054-024-04884-5 (PMC10988801; doi:10.1186/s13054-024-04884-5)
Supplement: Supplementary file 1 — Additional file 1. Figure S1. PRISMA Flow Diagram of Literature Search. Table S1. Anonymised Individual Panellist Scoring: Initial Assessment and Management of HF-CS. Table S2. Anonymised Individual Panellist Scoring: Escalation to tMCS in HF-CS. Table S3. Anonymised Individual Panellist Scoring: Weaning of tMCS in HF-CS. Table S4. Median Scores by Geographical Location (Europe vs North America). [file 13054_2024_4884_MOESM1_ESM.pdf]

SUPPLEMENTARY MATERIAL

Supplemental Figure

Figure S1 – PRISMA Flow Diagram of Literature Search

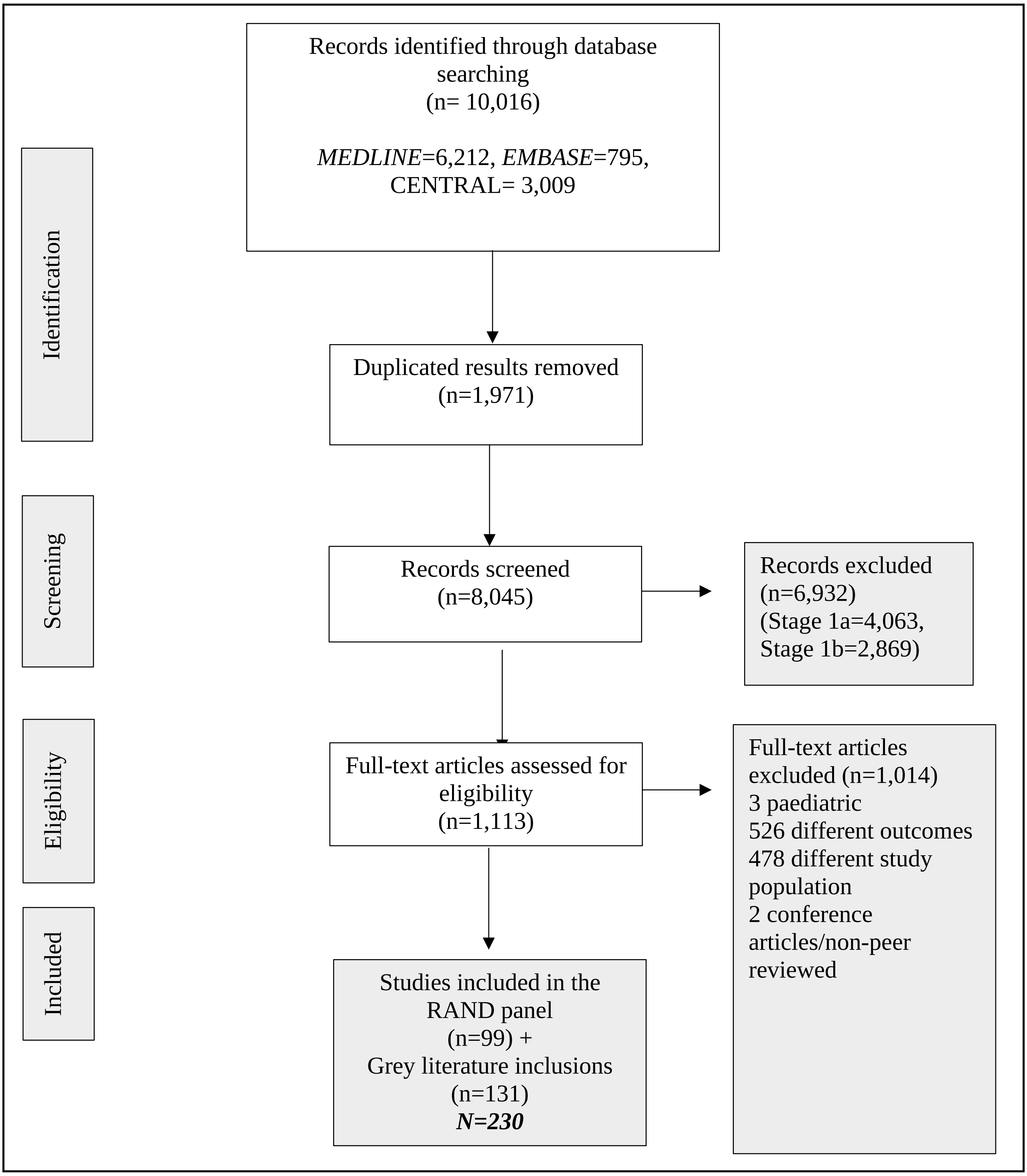

Supplemental Tables

Table S1 – Anonymised Individual Panellist Scoring: Initial Assessment and Management of HF-CS

Statement	Individual Panellist Scores (median in bold)	RAND Panel Outcome
**Please rate the appropriateness of the following in the initial assessment and management of SCAI Stage B/C HF-CS:**		
Focussed Cardiac Ultrasound	7-7-8-8-8-8-9-**9-9**-9-9-9-9-9-9-9	Appropriate
Pulmonary artery catheter	3-4-5-5-5-5-6-**6-6**-7-7-7-7-8-8-9	Uncertain
Point of care thoracic ultrasound	2-2-2-3-3-4-4-**5-5**-6-6-7-7-7-8-9	Uncertain
Point of care Abdominal Ultrasound	1-2-2-2-3-3-3-**3-4**-4-4-5-5-7-7-8	Uncertain
Norepinephrine as 1st line vasopressor	1-5-5-7-7-7-7-**7-7**-8-8-8-8-9-9-9	Appropriate
Dopamine as 1st line vasopressor	1-1-1-2-3-3-3-**3-4**-4-5-5-5-5-6-6	Uncertain
Dobutamine as 1st line inotrope	1-4-5-5-5-6-6-**6-7**-7-7-7-8-8-8-9	Appropriate
Milrinone as 1st line inotrope	1-4-4-5-5-5-5-**6-6**-7-7-7-7-8-8-9	Uncertain
Shock team discussion in patients suitable for escalation to tMCS	2-5-7-7-7-7-7-**8-8**-9-9-9-9-9-9-9	Appropriate
Application of prognostic scoring tools e.g. IHVI and CardShock to inform management and escalation	1-2-3-3-3-3-4-**4-4**-5-5-5-5-6-8-8	Uncertain

For each question, median scores were allocated as inappropriate if scoring <3.5, uncertain if ≥3.5 and <6.5 uncertain and appropriate if ≥6.5. DI was calculated using the RAND DI and disagreement deemed if DI ≥1 amongst the panellists.

HF-CS, Heart Failure related Cardiogenic Shock; IHVI, Inova Heart and Vascular Institute; SCAI, The Society of Cardiovascular Angiography and Interventions; tMCS, temporary Mechanical Circulatory Support.

Table S2 – Anonymised Individual Panellist Scoring: Escalation to tMCS in HF-CS

Statement	Individual Panellist Scores (median in bold)	RAND Panel Outcome
**Regarding the use of clinical, biochemical and haemodynamic parameters to guide escalation to tMCS in the context of maximal or optimal pharmacotherapy, please rate the appropriateness of the following:**		
Failure to achieve adequate diuresis / clinical decongestion	5-6-6-6-7-7-7-**7-8**-8-8-8-8-8-8-9	Appropriate
Lactate clearance	4-7-7-7-7-8-8-**8-8**-8-8-8-9-9-9-9	Appropriate
Serial worsening of liver function tests (bilirubin, transaminases & INR)	5-6-6-6-7-7-7-**7-8**-8-8-8-8-8-9-9	Appropriate
Serial worsening of renal function (urine output, creatinine, eGFR)	4-5-5-6-6-7-7-**7-7**-8-8-8-8-9-9-9	Appropriate
Serial worsening of central venous oxygen saturations ($ScVO_2$)	5-5-6-6-6-7-7-**7-7**-7-8-8-8-8-8-9	Appropriate
PAC haemodynamic data to inform escalation decisions	5-6-6-6-6-6-7-**7-8**-8-8-9-9-9-9-9	Appropriate
PAC haemodynamic data to inform device selection	5-5-6-6-7-7-7-**8-8**-8-8-9-9-9-9-9	Appropriate
Specific PAC thresholds (informed by AHA guidance, Geller et al.) to inform escalation decisions	3-3-3-3-4-4-4-**4-5**-5-5-6-6-6-7-8	Uncertain
Echocardiographic parameters to guide escalation decisions	3-4-5-5-5-5-5-**6-6**-6-6-7-8-8-8-9	Uncertain
Echocardiographic parameters to guide device selection	2-6-6-6-6-6-7-**7-7**-7-8-8-9-9-9-9	Appropriate
**Regarding the selection of tMCS in the management of SCAI Stage C HFCS, please rate the appropriateness of the following:**		
IABP as a tMCS option for bridge to recovery or durable therapies	1-3-3-4-4-5-5-**5-6**-7-7-7-7-7-7-8	Uncertain
Impella CP as a tMCS option for bridge to recovery or candidacy for durable HF therapies	3-3-3-4-4-5-5-**5-5**-6-6-6-7-7-7-8	Uncertain
Impella 5.0/5.5 as a tMCS option for bridge to recovery or candidacy for AHF therapies	3-5-6-6-6-6-7-**7-7**-7-7-7-8-8-8-8	Appropriate
Routine mechanical LV decompression in the context of peripheral V-A ECMO	2-3-3-4-5-5-6-**6-7**-7-7-8-8-8-8-8	Appropriate
Optimised pharmacological LV decompression prior to mechanical LV decompression	2-5-6-6-6-6-7-**7-7**-7-8-8-8-8-8-9	Appropriate
IABP as a mechanical LV decompression strategy in peripheral V-A ECMO	1-2-4-4-5-5-5-**5-6**-6-6-6-6-6-7-8	Uncertain
Impella (CP/5.0/5.5) as a mechanical LV decompression strategy in peripheral V-A ECMO	5-5-5-6-6-6-6-**6-7**-7-7-8-8-8-8-9	Appropriate

For each question, median scores were allocated as inappropriate if scoring <3.5, uncertain if ≥3.5 and <6.5 uncertain and appropriate if ≥6.5. DI was calculated using the RAND DI and disagreement deemed if DI ≥1 amongst the panellists.

AHA, American Heart Association; AHF, Advanced Heart Failure; eGFR, estimated Glomerular Filtration Rate; HF, Heart Failure; HF-CS, Heart Failure related Cardiogenic Shock; IABP, Intra-aortic Balloon Pump; Impella™ CP, Impella™ Central Pump; INR, International Normalised Ratio; LV, Left Ventricle; PAC, Pulmonary Artery Catheterisation; SCAI, Society for Cardiovascular Angiography and Interventions; $ScVO_2$, Systemic Central Venous Oxygen Levels; tMCS, temporary Mechanical Circulatory Support; V-A ECMO, Venoarterial Extracorporeal Membrane Oxygenation.

Table S3 – Anonymised Individual Panellist Scoring: Weaning of tMCS in HF-CS

Statement	Individual Panellist Scores (median in bold)	RAND Panel Outcome
**Regarding the weaning of tMCS in HF-CS, please rate the appropriateness of the following:**		
Routine PAC to assess / support weaning of tMCS	4-5-6-7-7-7-7-**7-7**-7-7-8-8-9-9-9	Appropriate
At least one attempt to wean tMCS before decision to transition to AHF therapies	2-5-6-7-7-7-7-**7-8**-8-8-9-9-9-9-9	Appropriate
Routine echocardiogram to assess / support weaning of tMCS	3-4-4-7-7-7-7-**7-7**-8-8-8-9-9-9-9	Appropriate
Use of Levosimendan to support weaning of tMCS	1-1-1-2-2-3-4-**4-5**-5-5-5-6-8-8-8	Uncertain
Use of escalating inotropes to wean from tMCS	1-3-3-5-5-6-6-**6-6**-7-7-7-7-8-8-9	Uncertain
Use of intravenous vasodilators to support weaning from tMCS	1-4-4-5-5-6-6-**6-7**-7-7-7-7-8-8-9	Appropriate
Trial of endothelin receptor antagonists or phosphodiesterase inhibitors in patients with evidence of pulmonary hypertension to support weaning from tMCS	3-3-3-3-3-4-4-**5-5**-5-5-5-6-6-6-7	Uncertain

For each question, median scores were allocated as inappropriate if scoring <3.5, uncertain if ≥3.5 and <6.5 uncertain and appropriate if ≥6.5. DI was calculated using the RAND DI and disagreement deemed if DI ≥1 amongst the panellists.

HF-CS, Heart Failure related Cardiogenic Shock; PAC, Pulmonary Artery Catheterisation; tMCS, temporary Mechanical Circulatory Support.

Table S4 – Median Scores by Geographical Location (Europe vs North America)

Statement	Overall Median	Europe (n= 9)	North America (n= 7)
**Please rate the appropriateness of the following in the initial assessment and management of SCAI Stage C HF-CS:**			
Focussed Cardiac Ultrasound	9	9	9
Pulmonary artery catheter	6	5	7
Point of care thoracic ultrasound	5	6	3
Point of care Abdominal Ultrasound	3.5	4	3
Norepinephrine as 1st line vasopressor	7	7	8
Dopamine as 1st line vasopressor	3.5	3	5
Dobutamine as 1st line inotrope	6.5	6	7
Milrinone as 1st line inotrope	6	6	7
Shock team discussion in patients suitable for escalation to tMCS	8	8	9
Application of prognostic scoring tools e.g. IHVI and CardShock to inform management and escalation	4	3	5
**Regarding the use of clinical, biochemical and haemodynamic parameters to guide escalation to tMCS in the context of maximal or optimal pharmacotherapy, please rate the appropriateness of the following:**			
Failure to achieve adequate diuresis / clinical decongestion	7	7	8
Lactate clearance	8	8	8
Serial worsening of liver function tests (bilirubin, transaminases & INR)	7.5	7	8
Serial worsening of renal function (urine output, creatinine, eGFR)	7	7	8
Serial worsening of central venous oxygen saturations (ScVO2)	7	7	7
PAC haemodynamic data to inform escalation decisions	7.5	7	9
PAC haemodynamic data to inform device selection	8	7	9
Specific PAC thresholds (informed by AHA guidance, Geller et al.) to inform escalation decisions	4.5	4	6
Echocardiographic parameters to guide escalation decisions	6	6	6
**Regarding the selection of tMCS in the management of SCAI Stage C HFCS, please rate the appropriateness of the following:**			

IABP as a tMCS option for bridge to recovery or durable therapies	5.5	4	7
Impella CP as a tMCS option for bridge to recovery or candidacy for durable HF therapies	5.0	6	5
Impella 5.0/5.5 as a tMCS option for bridge to recovery or candidacy for AHF therapies	7.0	7	7
Routine mechanical LV decompression in the context of peripheral VA ECMO	6.5	7	6
Optimised pharmacological LV decompression prior to mechanical LV decompression	7.0	7	7
IABP as a mechanical LV decompression strategy in peripheral V-A ECMO	5.5	5	6
Impella (CP/5.0/5.5) as a mechanical LV decompression strategy in peripheral V-A ECMO	6.5	7	6
**Regarding the weaning of tMCS in HFCS, please rate the appropriateness of the following:**			
Routine PAC to assess / support weaning of tMCS	7	7	7
At least one attempt to wean tMCS before decision to transition to AHF therapies	7.5	8	7
Routine echocardiogram to assess / support weaning of tMCS	7	8	7
Use of Levosimendan to support weaning of tMCS	4.5	5	3
Use of escalating inotropes to wean from tMCS	6	6	7
Use of intravenous vasodilators to support weaning from tMCS	6.5	6	7
Trial of endothelin receptor antagonists or phosphodiesterase inhibitors in patients with evidence of pulmonary hypertension to support weaning from tMCS	5	5	5

For each question, median scores were allocated as inappropriate if scoring <3.5, uncertain if ≥3.5 and <6.5 uncertain and appropriate if ≥6.5. DI was calculated using the RAND DI and disagreement deemed if DI ≥1 amongst the panellists.

AHA, American Heart Association; AHF, Acute Heart Failure; eGFR, estimated Glomerular Filtration Rate; HF, Heart Failure; HF-CS, Heart Failure related Cardiogenic Shock; IABP, Intra-aortic Balloon Pump; IHVI, Inova Heart and Vascular Institute; Impella™ CP, Impella™ Central Pump; INR, International Normalised Ratio; LV, Left Ventricle; PAC, Pulmonary Artery Catheterisation; SCAI, Society for Cardiovascular Angiography and Interventions; SCAI, The Society of Cardiovascular

Angiography and Interventions; $ScVO_2$, Systemic Central Venous Oxygen Levels; tMCS, temporary Mechanical Circulatory Support; V-A ECMO, Venoarterial Extracorporeal Membrane Oxygenation.
